# Nonadiabatic Forward Flux Sampling for Excited-State Rare Events

**DOI:** 10.1021/acs.jctc.2c01088

**Published:** 2023-03-01

**Authors:** Madlen Maria Reiner, Brigitta Bachmair, Maximilian Xaver Tiefenbacher, Sebastian Mai, Leticia González, Philipp Marquetand, Christoph Dellago

**Affiliations:** † Research Platform on Accelerating Photoreaction Discovery (ViRAPID), University of Vienna, Währinger Strasse 17, 1090 Vienna, Austria; ‡ Vienna Doctoral School in Physics, University of Vienna, Boltzmanngasse 5, 1090 Vienna, Austria; § Vienna Doctoral School in Chemistry, University of Vienna, Währinger Strasse 42, 1090 Vienna, Austria; ∥ Institute of Theoretical Chemistry, Faculty of Chemistry, University of Vienna, Währinger Strasse 17, 1090 Vienna, Austria; ⊥ Faculty of Physics, University of Vienna, Kolingasse 14-16, 1090 Vienna, Austria

## Abstract

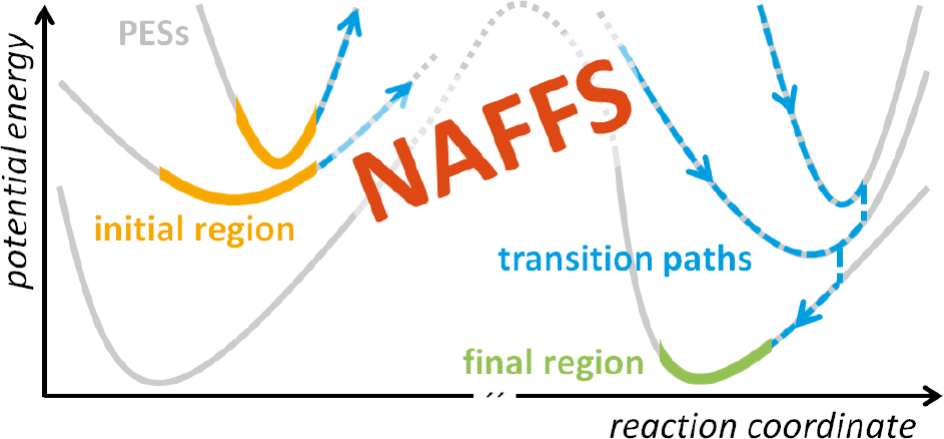

We present a rare event sampling scheme applicable to coupled electronic excited states. In particular, we extend the forward flux sampling (FFS) method for rare event sampling to a nonadiabatic version (NAFFS) that uses the trajectory surface hopping (TSH) method for nonadiabatic dynamics. NAFFS is applied to two dynamically relevant excited-state models that feature an avoided crossing and a conical intersection with tunable parameters. We investigate how nonadiabatic couplings, temperature, and reaction barriers affect transition rate constants in regimes that cannot be otherwise obtained with plain, traditional TSH. The comparison with reference brute-force TSH simulations for limiting cases of rareness shows that NAFFS can be several orders of magnitude cheaper than conventional TSH and thus represents a conceptually novel tool to extend excited-state dynamics to time scales that are able to capture rare nonadiabatic events.

## 1 Introduction

Chemical reactions initiated by the absorption of a photon are at the core of organic synthesis,^1^ catalysis,^2^ optogenetics,^3^ protein modification,^4^ conversion, and storage of solar energy^5^ and hold promise for many other applications.^6^ While many photochemical reactions are ultrafast and occur on a femtosecond time scale,^7^ high barriers on electronically excited potential energy surfaces (PESs) or nonadiabatic transitions^8^ with small couplings may lead to much slower reactions. For instance, the average reaction time for the keto–enol phototautomerism of 2-benzylbenzophenone is 0.5 ms,^9,10^ i.e., many orders of magnitude longer than the time scale of basic molecular motions. General classes of photoinduced processes of high relevance with possibly long time scales are electron transfer,^11^ photoinduced (pre)dissociation,^12,13^ and intersystem crossing.^14^

The resulting separation of time scales represents a huge challenge for the computer simulation of such rare reactive events. In particular, the femtosecond time step^15^ needed to accurately capture nuclear dynamics^16,17^ makes it unfeasible to simulate rare photoreactions using straightforward quantum dynamics^18,19^ or nonadiabatic mixed quantum–classical molecular dynamics,^20,21^ even if recently developed machine learning approaches bring such simulations from the picosecond^22^ to the nanosecond time scale.^23−27^

For classical dynamics in the electronic ground state, numerous computational methods have been developed to address the rare event problem,^16^ including umbrella sampling,^28^ blue-moon sampling,^29^ steered MD,^30^ hyperdynamics,^31^ milestoning,^32^ metadynamics,^33^ the string method,^34^ transition path sampling (TPS),^35^ and forward flux sampling (FFS).^36−38^ The investigation of rare events in excited-state problems, however, is much less explored. Recent efforts relied on metadynamics to probe intersection crossing points between adiabatic electronic states that lead to the slow formation of photoproducts.^39−41^ Nonadiabatic rare event simulation methods based on a generalization of transition-state theory of infrequent events to excited states were developed.^15,42,43^ In other work, TPS was used to sample the nonadiabatic dynamics of open quantum systems as described by a quantum master equation preserving detailed balance.^44^ TPS has also been applied to semiclassical pathways^45^ obtained by trajectory surface hopping (TSH).^46^ In contrast to other rare event methods, TPS does not require any prior knowledge of the reaction mechanism in terms of a reaction coordinate. The backward propagation of trajectories required in TPS, however, is not possible in the framework of TSH.^47^ While this difficulty can be circumvented by generating reverse trajectories with approximate quantum weights and subsequently reweighting them,^45,48^ this procedure reduces the efficiency of the TPS simulation.

In this paper, we show how rare but important events occurring in electronic excited states can be studied with FFS, a trajectory-based approach originally developed for driven nonequilibrium stochastic systems with unknown stationary phase space distribution. In this approach, a sequence of nonintersecting interfaces between reactants and products is used to sample the ensemble of transition paths and calculate reaction rate constants. We combine the FFS methodology with TSH dynamics, exploiting that FFS requires only forward integration of the equations of motion. Hence, it is not affected by the lack of time reversal symmetry of TSH. As demonstrated using two illustrative models (an avoided crossing and a conical intersection, two ubiquitous features of nonadiabatic PESs), the novel nonadiabatic FFS (NAFFS) method presented here provides a general approach for enhanced path sampling in electronic excited states. NAFFS also allows studying rare nonadiabatic reactions on time scales exceeding by far those accessible with brute-force TSH simulations.

The remainder of the paper is organized as follows. In section 2, we lay out the FFS algorithm and describe how it is combined with TSH. Details on the implementation of the method are provided in section 3. Results obtained for two simple model systems are presented and discussed in section 4, and conclusions are provided in section 5.

## 2 Theory

In the following, we review the main concepts behind the FFS method and the TSH algorithm and explain how they had to be extended in order to be combined into NAFFS.

### 2.1 Forward Flux Sampling of Rare Events in Electronically Excited States

Rare event sampling methods for ground-state problems sample regions of the phase space that are unlikely to be visited by standard MD calculations. Among them, TPS approaches sample fully dynamical trajectories (i.e., trajectories which could occur in exactly the same way in brute-force MD simulations with the correct probability) with Monte Carlo methods acting in path space (i.e., trajectory space).^16^ In these algorithms, transition paths (i.e., rare trajectories that start in the initial reactant region of phase space and end in the final product region of phase space) are sampled by generating a new trajectory from a given trajectory, typically by propagating the system both forward and backward in time. The newly generated trajectory is then accepted or rejected according to a criterion guaranteeing that the trajectories follow the statistics dictated by the transition path ensemble. In this way, reactivity is maintained at all times during the TPS simulation and no computing resources are wasted to follow the dynamics of the system during the long periods when no transition occurs. Analysis of the sampled transition paths then provides insights into the underlying reaction mechanisms.^35^ In transition interface sampling,^49^ a variant of TPS, ensembles of pathways that cross a sequence of interfaces between reactant and product regions are considered. This procedure significantly enhances the sampling of transition paths and uses the rejected trajectories ingeniously to calculate reaction rate constants.

As in most other TPS methods, transition interface sampling also relies on the time reversibility of the dynamics and on explicit knowledge of the stationary phase space distribution. Hence, the application of TPS methods to irreversible nonequilibrium systems is not straightforward. This difficulty motivated the development of FFS,^36−38,50,51^ a simulation method for rare events in which the equations of motion are integrated only forward in time such that it does not suffer from lack of knowledge of the stationary distribution and the absence of microscopic reversibility. Hence, FFS, a splitting method based in spawning swarms of trajectories connecting interfaces, can be applied to study rare events occurring in nonequilibrium systems.^50,52^ These properties make FFS ideally suited for combining it with TSH, which does not satisfy detailed balance and generally produces an unknown stationary distribution.

**Figure 1:**
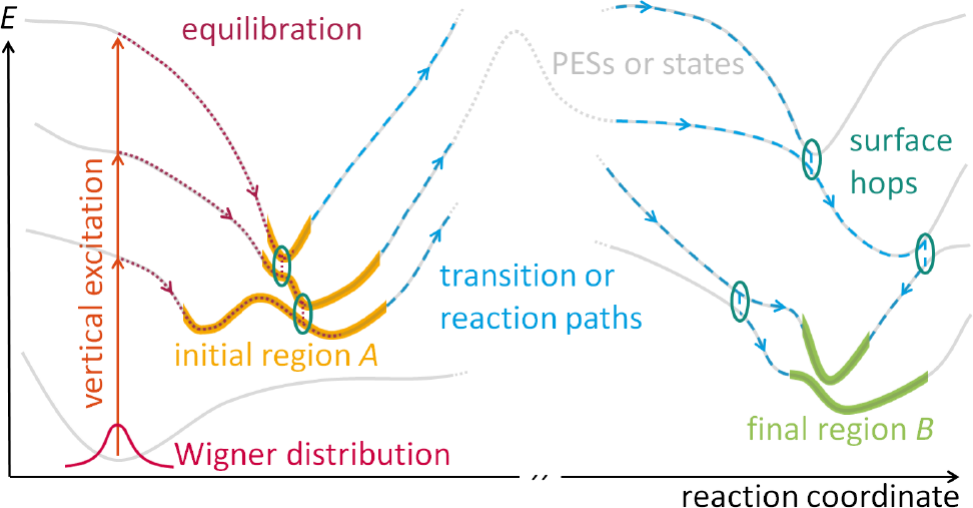
Schematic illustration of a nonadiabatic PES landscape, showing a possible definition of the initial and final regions *A* and *B*. In the framework of a typical TSH simulation, a nuclear ensemble of vertically excited configurations drawn from the Wigner distribution relaxes into one or several minima included in *A* (equilibration), which serves as the starting region for NAFFS. NAFFS then samples reaction paths that connect *A* and *B* including rare events in electronic excited states (e.g., an energy barrier). Due to the nonadiabatic nature of the PES landscape, surface hops (circles) between states can happen during the dynamics.

As it is customary in FFS, we consider systems that undergo a rare transition from an initial reactant region of phase space called *A* to a final product region called *B*. Both regions, *A* and *B*, are supposed to be stable, meaning that the system resides in them for long times compared to the time when it is in an unstable region, e.g., when it undergoes a transition from *A* to *B*.^16^ Both regions are defined in terms of collective variables, that is, functions of the phase space coordinates, e.g., bond lengths, angles, or dihedrals.^53^ What distinguishes our application of FFS from others is the novelty that we explicitly include one or several PESs in the definition of our initial and final regions (see Figure 1); this is necessary to study nonadiabatic excited-state reactions. Throughout this work, we use the term “region” for stable phase space regions in the FFS context and the term “transition path” as a synonym of “reaction path” to describe the evolution of the system between regions. We use the term “state” for electronic states or PESs and the term “hop” to describe nonadiabatic changes between states. Hence, in our nonadiabatic setting, one or several states can be part of the definition of a region, and a transition path between regions can include hops between electronic states.

A typical TSH simulation begins with an instantaneous vertical excitation (that mimics the absorption of a photon) of a nuclear ensemble of geometrical configurations, e.g., drawn from a Wigner distribution in the electronic ground-state minimum (see Figure 1, left).^21^ In excited-state reactions involving rare events, after the vertical excitation the system would evolve (see dark red dotted equilibration trajectories in Figure 1) into a stable excited-state region *A*, in which it stays for a very long time before the rare event occurs and the system transitions to the final region *B* (see blue dashed transition paths in Figure 1). To ensure the greatest possible generality in the choice of reaction pathways, the stable excited-state region *A* may span several PESs, where the different configurations of the nuclear ensemble land after equilibration. Further, the initial region *A* is flexible enough to include different regions of a single PES, if needed. Likewise, the final region *B* (see green PESs parts in Figure 1) can expand over multiple states. For simplicity, neither the vertical excitation process nor the equilibration to the initial region *A* is included in our NAFFS algorithm but can be performed with standard initial condition and TSH simulations.

As the transition interface sampling method,^49^ FFS is an interface-based approach^54^ where interfaces λ_*i*_ (i.e., intermediate stages between regions *A* and *B*) are defined in terms of collective variables. There exist several approaches to the proper placement of the interfaces.^55−58^ In NAFFS, the interfaces are also able to include a range of PESs, as the initial and final regions do. For the applications presented later, we define the first and last interfaces to equal the boundaries of the stable regions *A* and *B*, i.e., λ_*A*_ ≡ λ_0_ and λ_*B*_ ≡ λ_*n*+1_, as often done so.^38^ The rate constant *k*_*AB*_ is then calculated as^36^
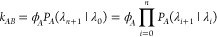
1where ϕ_*A*_ is the effective positive flux out of *A* through the boundary of *A* and *P*_*A*_(λ_*j*_|λ_*i*_) is the probability of a trajectory that started in *A* crossing the interface λ_*j*_ in the direction of *B* given that it has already crossed the interface λ_*i*_.

**Figure 2:**
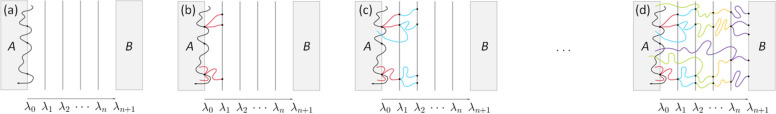
Working principle of the forward flux sampling scheme.^50^ (a) Brute-force MD simulation in the initial reactant region *A* is performed, and snapshots where the trajectory exits *A* are stored as initial shooting points. (b) Shots of randomly chosen initial shooting points are either rejected if they enter *A* or accepted if they cross the adjacent interface λ_1_. Due to the underlying stochastic dynamics, trajectories initiated in the same shooting point differ. Crossing snapshots are stored as shooting points for the next FFS cycle. (c) In the second FFS cycle, randomly chosen trajectories ending in shooting points on the interface λ_1_ are continued and either accepted if they cross the next adjacent interface or rejected if they enter *A*. This procedure is repeated for all interfaces λ_*i*_. (d) Once the last FFS cycle is finished, final transition paths from *A* to *B* are obtained by piecing together the accepted partial paths obtained in each FFS cycle.

There are two common ways to calculate the flux ϕ_*A*_ through the boundary of the initial region *A*. The first,^57^ later referred to as “flux with reset”, involves running an MD simulation in region *A* and counting the number of times *N*_0_ that the interface λ_0_ is crossed in the outward direction of *A* divided by the simulation time (see Figure 2a). Here, it is assumed that the trajectory does not enter the final product region *B* during the MD simulation or if it does it is immediately reset to *A* and re-equilibrated.^37^ Alternatively, using a second method—later referred to as “flux without reset”—only for equilibrium systems and reversible reactions and if the trajectory visits both regions *A* and *B* several times in the MD run, the flux ϕ_*A*_ can be calculated by dividing the number *N*_0_ by the time  the system has spent in the overall region  during the simulation^49^
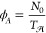
2The time  spent in the overall region  is not only the time the trajectory is located in region *A* but also the time spent outside *A* as long as *B* is not reached. If the trajectory enters *B*,  resumes counting after the trajectory exits *B* and re-enters *A*. Provided that both approaches to calculate the flux ϕ_*A*_ are applicable, one or the other could be computationally more efficient and which one to take is decided depending on the system to study.

The crossing probabilities *P*_*A*_(λ_*i*+1_|λ_*i*_) are given by the fraction of accepted Monte Carlo moves or “shots” that are initiated on the shooting interface λ_*i*_ and cross the subsequent interface λ_*i*+1_ with respect to the total number *M*_*i*_ of trial shots initiated from λ_*i*_
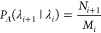
3where *N*_*i*+1_ is the number of accepted shots which reached interface λ_*i*+1_. In the first FFS cycle, the shooting points on the boundary λ_0_ of the initial region *A* are randomly chosen from the *N*_0_ crossing points collected in the flux simulation, exploiting the fact that due to the underlying stochastic dynamics of the system (induced typically by a thermostat), two shots beginning in the same point produce different trajectories (see Figure 2b). Final points of accepted shots in each FFS cycle serve as possible shooting points for the next FFS cycle (see Figure 2c).^37^ Final reactive paths (see Figure 2d) are obtained in accordance to their correct weight in the transition path ensemble, i.e., the relative probabilities of transition paths with respect to the considered system; hence, those of reactive paths obtained in MD simulations are conserved.^59^

In summary, the difficult problem of finding a reaction coordinate and a phase space probability distribution is replaced by the (usually) simpler task of defining reactant and product regions *A* and *B* and interfaces in between. In a general excited-state application, MD simulations starting in reactant and product might provide hints about suitable definitions of *A* and *B*. Further, it can be useful to look at which region the system equilibrates to after excitation in order to find a suitable initial region *A* (see Figure 1). One could use constrained dynamics and employ scans along selected collective variables or compute minimum crossing points to find appropriate interface placements. Note that the interfaces do not have to be defined in advance, i.e., one can place interface λ_*i*+1_ after having performed the FFS cycle yielding trajectories reaching the previous interface λ_*i*_. Further, some approaches from ground-state FFS on how to define interfaces could be extended to NAFFS.^57,60^

These considerations, however, affect the application of the FFS method and not its implementation (see section 3).

The relative error of the rate constant *k*_*AB*_ obtained in an FFS simulation can be estimated as^38,57,61^
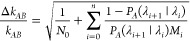
4where Δ*k*_*AB*_ is the standard deviation of the calculated rate constant *k*_*AB*_ and *M*_*i*_ is the number of shots performed starting from shooting points on interface λ_*i*_. The relative error estimation given by eq 4 corresponds to a Gaussian error propagation, taking into consideration an estimate of the relative error of the flux ϕ_*A*_ as
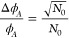
5and the estimated errors of the crossing probabilities obtained for each shooting interface as
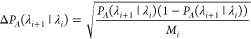
6

### 2.2 Stochastic Excited-State Molecular Dynamics Simulations Using Trajectory Surface Hopping

The application of the FFS method requires an MD algorithm to propagate the system in time. Here, we use a velocity Verlet-type^62−64^ algorithm with Langevin dynamics in combination with the surface hopping including arbitrary couplings (SHARC) approach.^65−67^ SHARC is an extension of Tully’s fewest switches^68^ TSH method, able to describe on the same footing internal conversion between states of the same spin multiplicity via nonadiabatic couplings and intersystem crossing between states of different spin multiplicity via spin–orbit couplings.

As a TSH method, SHARC is a mixed quantum–classical simulation technique, where the nuclei are considered classical particles and nonadiabatic effects are accounted for by including multiple PESs.^69^ Nuclei always follow the force corresponding to one single PES (the “active state”), and instantaneous hops between adiabatic PESs mimic nonadiabatic changes, according to hopping probabilities based on the quantum mechanical evolution of the electronic populations of the different states.^67^ As the TSH algorithm treats the electrons quantum mechanically, it solves the electronic time-dependent Schrödinger equation^66^
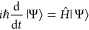
7where *Ĥ* is the electronic Hamilton operator, *ℏ* the reduced Planck constant, and |Ψ⟩ the electronic wave function, which in a linear combination of basis states ψ_α_ is written in terms of the coefficients *c*_α_
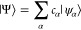
8Combining eqs 7 and 8 yields the equations of motion for the electronic population vector **c** consisting of the electronic wave function coefficients *c*_α_
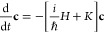
9with coupling matrix *K* and Hamilton matrix *H*.

SHARC uses a fully “adiabatic” or diagonal representation in the propagation.^65,66^ In the simulations presented in this work, the coefficients *c*_α_ are propagated as^67^
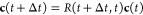
10with time step Δ*t*. The corresponding propagator matrix *R* is given as
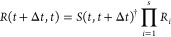
11with
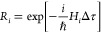
12and
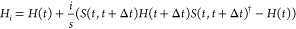
13where *s* is the number of substeps with length Δτ = Δ*t*/*s* within a time step of length Δ*t* and overlap matrix *S* is calculated by *S*(*t*, *t* + Δ*t*) = *U*^†^(*t*)*U*(*t* + Δ*t*) from transformation matrices *U* obtained by diagonalizing the diabatic Hamiltonian *H* = *U*^†^*H*^diab^*U* at times *t* and *t* + Δ*t*. The overlap matrix and Hamiltonian are phase corrected^67^ in each time step to avoid random changes in the population transfer direction because of random phases of the transformation matrices *U*. The hopping probabilities, i.e., the probabilities to hop from the current or active state β to a different state α, are given by^66^
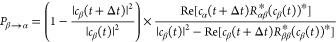
14with electronic coefficients in states α and β, namely *c*_α_ and *c*_β_, and complex conjugated elements of the propagator matrix *R*. A surface hop to state α̃ is attempted only if a random number *r* drawn from a uniform distribution in the interval [0,1] satisfies^70^
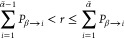
15If the total energy of the system is smaller than the potential energy of the envisaged new state α̃, no hop is performed and the system stays in state β, i.e., the new active state β is the same as the old state—this is called a “frustrated hop”.

Otherwise, if the potential energy  of the envisaged new state α̃ is smaller than or equal to the system’s total energy *E*_total_, a surface hop β→α̃ is performed. As in most TSH implementations, during the surface hop the total energy is kept constant by rescaling the nuclear velocities **v**. By default in SHARC, this is done according to the scheme^21^
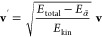
16where *E*_kin_ is the total kinetic energy before the hop, i.e., the rescaled velocity vector **v**′ is parallel to the original one, **v**. After this adjustment, the state α̃ is the new active state β of the system. Other velocity adjustment varieties are available in SHARC.^71^

In TSH, electronic populations in the nonactive states α follow the forces of the active state β, even though in a proper quantum mechanical description they should follow the forces of their respective state α. This problem is known as “overcoherence”^20,21,72^ and in the present work is accounted for by modifying in every simulation time step the electronic coefficients of the states α according to the energy difference to the active state β after the surface hopping procedure^73^
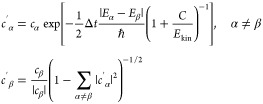
17with decoherence parameter *C*. This approach collapses the amplitudes of the nonactive states outside of a crossing region, because amplitudes of nonactive states should follow different trajectories. The time scale of this amplitude collapse (the decoherence time^74,75^) is modeled here using the “energy-based decoherence scheme”^73^ that is widely used in the surface hopping community.^20,21^

Although TSH algorithms already have an intrinsically stochastic character due to the randomness of the hops, their degree of stochasticity is not sufficient for an application of the FFS algorithm, especially in regions away from probable hopping points. These regions are characterized by large energy gaps between adjacent PESs. To increase the level of stochasticity beyond random hops in a controllable way, we consider a system evolving under the influence of friction and random forces as described by the Langevin equation
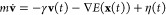
18Here, **x** denotes the positions of all atoms, *E* is the potential energy, *m* is the mass, and γ is the friction constant controlling the magnitude of the frictional forces, which are proportional to the velocities. In the above equation, η(*t*) denotes Gaussian white noise with zero mean, ⟨η(*t*)⟩ = 0, and delta-like correlations  with Boltzmann’s constant *k*_B_ and temperature *T*. The Langevin equation can be viewed as resulting from coupling the system to a heat bath with temperature *T* that causes friction and random forces related by the fluctuation–dissipation theorem. The strength of the coupling to the heat bath is controlled by the friction constant γ, and for γ = 0, the Langevin equation reduces to Newton’s equations of motion.

The Langevin equation in eq 18 is solved numerically in small time steps Δ*t* using a velocity Verlet-like integration scheme^76^
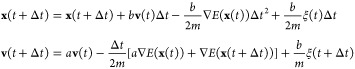
19where , and . In the above equations, ξ(*t* + Δ*t*) denotes a vector of independent Gaussian random numbers with zero mean and variance 2*γk*_B_*T*Δ*t*.

This methodology allows SHARC simulations to follow stochastic dynamics and is, therefore, ideally suited for combination with the FFS algorithm. Note that in TSH, hops are performed within one time step, which results in a hop either taking place or not. This means that partial hops cannot happen, which also means that we cannot place horizontal NAFFS interfaces between two states, i.e., during a hop. As a consequence, models of rare events where the rareness is solely due to a single hop between adiabatic surfaces, which occurs with a low probability and which the system must perform in order to undergo any transition, i.e., where the rareness is not related to nuclear motion reaching a certain region in phase space, would be beyond the applicability of NAFFS as defined here. Such a case is very unlikely in the high-dimensional PES landscapes of real systems, where multiple paths connect the reactant and product regions. Yet, it could, for example, be addressed by biasing the hopping probability and accordingly reweighting the results.^77^

## 3 Implementation

**Figure 3:**
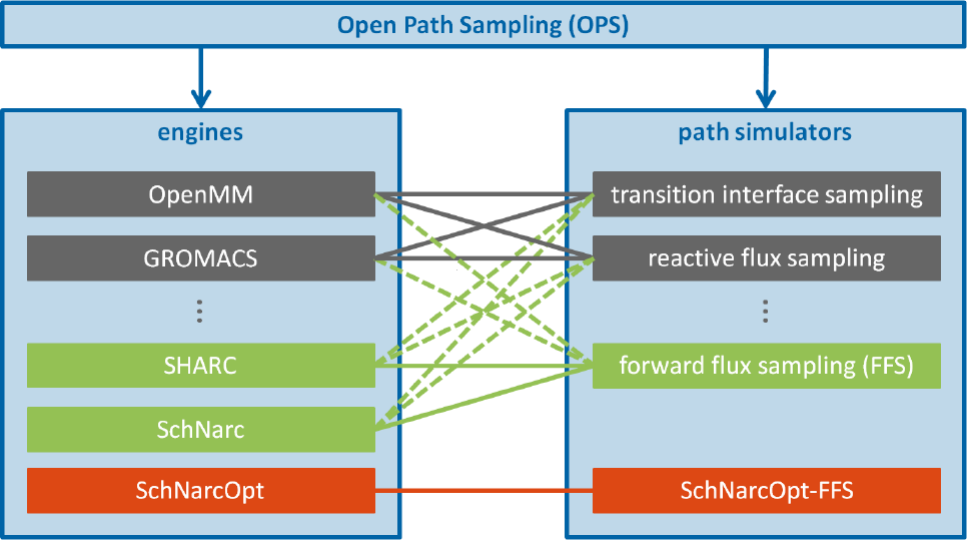
Architecture of the OPS library. Examples of existing engines, simulators, and combinations between them are shown in gray. Green boxes indicate new implementations. Dashed green lines indicate new possible combinations of engines and simulators and solid lines explicit new implementations of NAFFS. Dedicated SchNarcOpt engine tailored to a new FFS implementation SchNarcOpt-FFS for computational efficiency is highlighted in red.

The practical implementation of NAFFS relies on two program suites. On the TSH side, we extended SHARC^65−67^ with a Langevin thermostat to endow the dynamics with the stochasticity required for FFS (section 2.2). On the FFS side, we used Open Path Sampling (OPS),^78,79^ a Python library for path sampling simulations capable to work with various MD codes. For example, OPS is interfaced with two popular MD engines: OpenMM^80,81^ and GROMACS^82,83^ (see gray engines in Figure 3). We have now implemented the SHARC suite and the SchNarc^84^ method via the SHARC driver pySHARC^85^ as new general engines in OPS, see green engines in Figure 3. Hence, any quantum chemical method compatible with the SHARC program for computing PESs and electronic properties is now also available for NAFFS simulations. SchNarc was originally developed as an interface between the SHARC program and an extension of the neural network potential SchNet^86,87^ to excited-state properties.^84^ We do not use neural networks in this work, but because SchNarc is not based on file I/O, it allows for computationally much more efficient path sampling simulations using TSH dynamics. An additional advantage of the SchNarc engine is that it opens the possibility of using neural network potentials to compute PESs in the future, further decreasing computational costs.

To accompany both engines, SHARC and SchNarc, we implemented an OPS tool to capture snapshots, i.e., individual frames of a trajectory. Such a tool is needed to describe the system at a particular point in time, where specific ranges of PESs are included in addition to the atomic positions and velocities that define the collective variables. Both the ranges of PESs and the collective variables are used for the definition of the stable initial and final regions *A* and *B* as well as the definition of the FFS interfaces.

We note that, as usual in OPS, we also include in our engines an additional rejection criterion for discarding trial shots if those exceed a user-given number of time steps. Although this number should be chosen such that (almost) no trajectories are discarded, such implementation is useful as it aids, for example, the discovery of new stable minima in the region between *A* and *B*. From a practical point of view, a rejection criterion is also helpful to avoid very long unnecessary calculations in such a priori unknown stable minima by terminating the respective trajectory calculation and continuing with the next shot.

In OPS any engine can be combined with any TPS simulator, such as transition interface sampling^49^ or reactive flux sampling,^88^ see Figure 3. As an additional transition path sampling method in the OPS library, here we implemented a general FFS simulator applicable to excited states, see green simulator in Figure 3. This code (see section S1) solely includes the above-described procedure, where eqs 2 and 3 are relevant.

The advantage of both the general SHARC engines and the general FFS simulator is that they are implemented in the spirit of OPS, i.e., any engine can be combined with any path simulators, so that, in principle, now SHARC (or SchNarc) can be used with any method available in OPS (dashed green lines) and certainly both engines effectively work with FFS (solid green lines). Despite their flexibility, we have also created a dedicated SchNarcOpt engine that is combined with an optimized FFS-SchNarcOpt path simulator (red in Figure 3), which, although specific, is computationally more efficient since it does not require file I/O. This specific NAFFS implementation has been used in the applications below in order to keep the computational cost as low as possible.

The vertical excitation and the equilibration process (recall Figure 1) pose a necessary preliminary step prior to any NAFFS simulation. In this work, these steps are carried out in a plain SHARC/SchNarc-TSH simulation. Thus, the workflow of a NAFFS simulation begins with the flux simulation and collection of initial shooting points on the boundary of the initial region *A* (see Figure 2a). This process is done via the SchNarcOpt engine through OPS, and it is followed by the FFS cycles (see Figure 2b–d), executed via the SchNarcOpt-FFS path simulator in OPS.

In summary, the workflow of a typical NAFFS simulation starts with generating initial conditions (e.g., Wigner sampling and vertical excitation, see Figure 1) and a relaxation into the initial region, followed by the NAFFS method that generates transition trajectories between the initial and the final region.

To ensure transparency and reproducibility of the obtained results, the code developed is made freely available (see section S1).

## 4 Results and Discussion

As a first application of NAFFS, here we employ two dynamically relevant analytical potential energy landscapes that have been constructed to include rare events in different conditions. We deliberately choose simple analytical models for testing NAFFS rather than a real molecule because they allow for a systematic investigation of different parameters and demonstrate the broad applicability of NAFFS even in extreme situations, regardless of low or high temperature, strong or weak nonadiabaticity.

Further, our models can be tuned so that they can be studied under different conditions. Using these models, we show that NAFFS results agree quantitatively and qualitatively with reference brute-force TSH simulations on the very same models. These TSH calculations have also been performed via the SchNarcOpt engine through OPS, and they employ the same underlying dynamics (see section 2.2) and TSH protocol (see sections 4.1 and 4.2) as the NAFFS simulations we compare them against. By construction, the NAFFS methodology is independent of the underlying dynamics and of specifics of the employed TSH method. Note that in this work, we do not test different TSH protocols^20,21,89,90^ against each other, e.g., using different choices of decoherence schemes,^73,91−94^ velocity rescaling methods,^46,91^ hopping procedures,^65,95^ or velocity reversal on frustrated hops.^96^ In particular, we are aware that rescaling along the nonadiabatic coupling vector is generally more accurate; however, this paper is not about how accurate TSH is and which TSH protocol one should use for a given system. Our manuscript shows that NAFFS and brute-force TSH give consistent results using exactly the same models and TSH protocol. We also do not attempt to investigate the accuracy of TSH dynamics against more accurate quantum dynamics. Such topics have been extensively discussed in previous literature.^71,93,94,97,98^ The future simulation of real molecules is straightforward, i.e., does not require any further implementation, only investing in the calculation of on-the-fly multidimensional PESs at the desired quantum chemical level of theory.

The first system features an avoided crossing between two excited states, see section 4.1. Using this model, we demonstrate the essential functionality of NAFFS by calculating the temperature dependence of the transition rate constant with the energy barrier of the avoided crossing. Further, for this model, we investigate the influence of nonadiabatic effects on the transition rate constant by varying the gap size between the PESs and thus the rareness of the event.

The second system includes a conical intersection between two states, see section 4.2. This model shows richer nonadiabatic dynamics than the one-dimensional avoided crossing and allows us to focus on the dependence of the reaction rate constant on temperature and to study the contributions of trajectories with different numbers of hops.

Both models demonstrate that NAFFS yields correct results compared to reference plain brute-force TSH simulations in a fraction of the computational time and thus is ideally suited to study general rare nonadiabatic reactions.

### 4.1 Rare Event Dynamics through an Avoided Crossing

**Figure 4:**
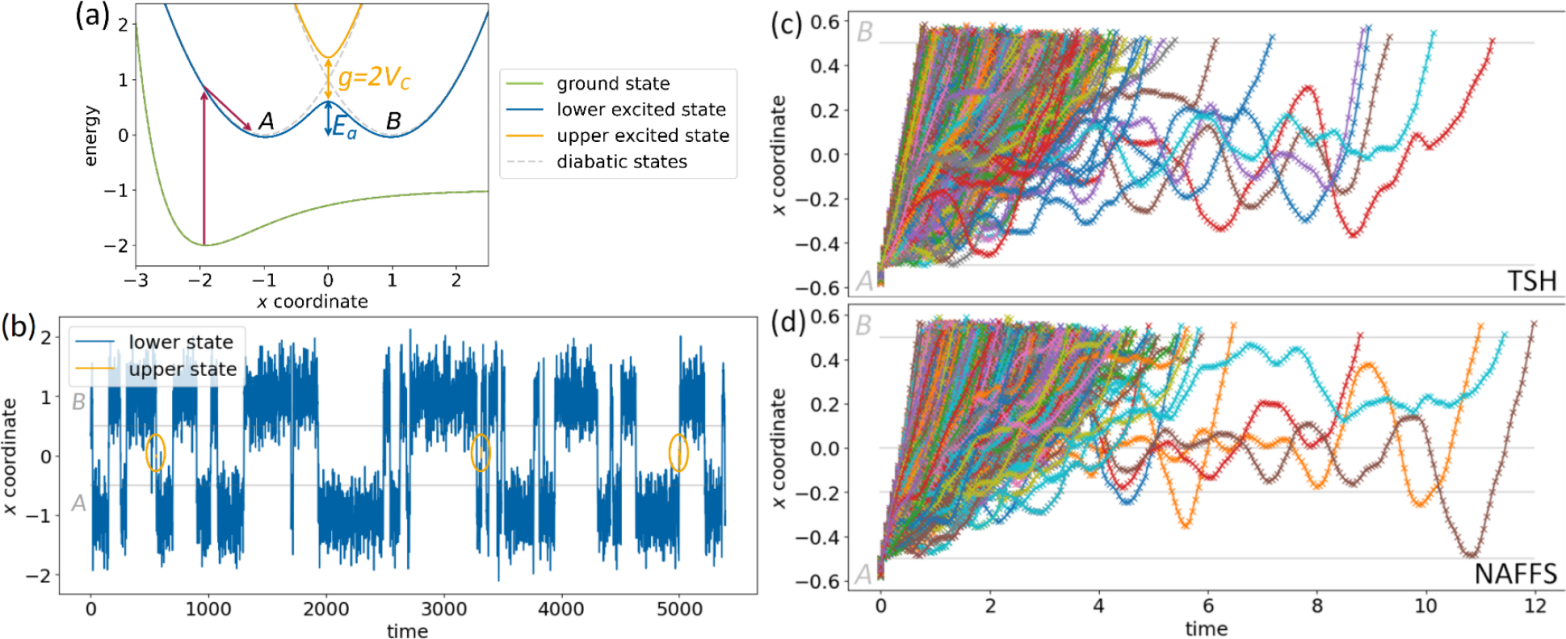
(a) Analytical model system consisting of an adiabatic ground state (green) and two adiabatic excited states (upper and lower), shown as solid lines, which avoid each other by a gap size *g* = 0.8. Lower state barrier height is denoted as *E*_a_. Corresponding diabatic PESs are shown as dashed lines. Schematically shown preprocessing (dark red, vertical excitation from the ground state and relaxation into the initial region *A*) is not part of the NAFFS routine (see section 2.1). (b) Representative cutout of a TSH trajectory propagated in this system as a function of time, where the first snapshot of the cutout is shifted to time zero for convenience. Trajectory parts that are spent in the upper state are shown in orange (and additionally marked by orange ovals) vs blue in the lower state. Boundaries of the initial and final regions *A* and *B* are depicted in gray. (c and d) Superimposed transition paths between the boundaries of the initial and the final regions (gray lines) from TSH (c) and NAFFS (d) simulations within this system with *V*_c_ = 0.4. Different colors allow discriminating individual transition paths; symbols indicate calculated time steps. Time of the first snapshot of each transition path is set to zero for convenience. Number of plotted transition paths is (c) 1069 (TSH) and (d) 1803 (NAFFS). In the latter case, interfaces are also plotted as gray lines. Note that for a better visualization, we only plot transition paths that are shorter than 12 time units (>99.5%); this leaves out 4 events in the case of TSH and 2 in the case of NAFFS (compare with Table 1).

We define a three-dimensional potential energy landscape from two excited diabatic harmonic potentials of the form
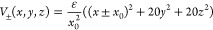
20defined in the Cartesian coordinates *x*, *y*, and *z*. The diabatic potentials are coupled to each other by a constant coupling of *V*_c_, giving the diabatic Hamiltonian
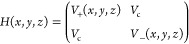
21which upon diagonalization provides the corresponding avoiding adiabatic potentials (here labeled as lower and upper states) and the gap *g* between them (see Figure 4a). Note that we also include an additional ground state in Figure 4 to clarify the mental connection to Figure 1. However, as the vertical excitation from the ground state and the equilibration to the initial region are standard tasks and not included in the NAFFS workflow (see section 2.1) but can be seen rather as a preprocessing step, we do not further discuss them here.

This simple model of two intersecting and coupled diabatic potentials is prototypical for a wide range of important photoinduced processes, e.g., electron transfer,^11^ photodissociation,^12,13^ or intersystem crossing.^14^

For the remainder of section 4.1, we will consider the two adiabatic excited states (labeled as lower and upper states).

In the *x* direction, the lower potential in adiabatic representation is bistable with minima at ±*x*_0_ separated by a barrier of height ε. The tight harmonic potentials acting on the *y* and *z* coordinates make the system effectively one dimensional along the *x* coordinate. In particular, the additive terms in *y* and *z* are invariant under a diagonalization of the diabatic Hamiltonian. However, the dynamics is coupled in all directions due to the velocity rescaling (see eq 16) and decoherence scheme (see eq 17); hence, we have some weak normal mode coupling in addition to the electronic coupling given by *V*_c_. Similar cases also appear commonly in nature, i.e., some degrees of freedom govern the nonadiabatic dynamics, while weakly coupled degrees of freedom hardly contribute. We use system-specific self-consistent units, i.e., we measure energies in units of ϵ, lengths in units of *x*_0_, and masses in units of *m*, where the mass of our system is 1 (see section S2.1 and Table S1) for the remainder of this section. Time is measured in units of .

This model of an avoided crossing is used to compare quantitative and qualitative results against plain brute-force TSH simulations which are performed using the same system. In this way, we demonstrate that NAFFS yields the correct reaction rate constants and evaluate its computational efficiency. Varying the temperature and the strength of the diabatic coupling gives insight into how these parameters fundamentally condition the transition rate constants.

For the first simulation we use *V*_c_ = 0.4, which is large compared to other choices of the coupling constant considered later. The resulting energy gap of this strongly avoided crossing is *g* = 0.8 (see Figure 4a). This rather large energy gap is expected to lead to predominantly adiabatic behavior.

Accordingly, one expects very few nonadiabatic surface hops in the generated trajectories and a dynamics that is predominantly adiabatic in the lower state potential with occasional hops to the excited state. The lower state resembles a one-dimensional double-well potential, where we define the lower state minimum around *x* = −1 as initial region *A* and the lower state minimum around *x* = 1 as final region *B*. The two stable regions are separated by a lower state energy barrier of *E*_a_ = 0.64, see Figure 4a.

All simulations presented in this section employ the default TSH decoherence parameter^73^ (*C* = 0.1 in au, see section S2.2) and *s* = 25 substeps (see eq 11). The remaining adjustable parameters are the temperature, the time step, and the friction constant. The time step is set to approximately Δ*t* = 0.0539, such that one oscillation in a lower state minimum consists of at least 20 time steps and hence the dynamics of the system can be adequately captured. The temperature is set to *T* ≈ 0.2133, such that the lower state barrier height is 3 *k*_B_*T*. This barrier height is sufficiently large to make the barrier crossing a rare event possible and, at the same time, sufficiently low to enable the observation of transition events in brute-force TSH simulations.

The rate constant of a transitions from *A* to *B* depends on the friction constant γ that appears in the Langevin equation (see eq 18).^99^ In particular, the rate constant shows a maximum as a function of the friction constant, known as the Kramers turnover.^99−104^

For smaller and larger values of γ, the rate constant decreases: at low friction because of slow energy exchange of the system with the environment and at high friction due to slow diffusion at the barrier top. The maximum transition rate constant is expected to occur close to the friction constant at which the energy dissipated as the system crosses the barrier is about 1 *k*_B_*T*. For a barrier shaped like an inverted parabola, this conditions implies γ_max_/*m* = *ωk*_B_*T*/*E*_a_, where γ_max_ is an estimate for the turnover friction and ω is the frequency of the unstable mode at the barrier top. For the parameters selected here, the turnover friction is γ_max_ ≈ 0.94. Hence, the friction coefficient selected for our simulations, γ = 1.4133, is slightly higher than the turnover friction. Alternatively, one could estimate the position of the Kramers turnover by considering the limit of validity of the high friction expression of the rate constant resulting from Kramers theory. This approach yields γ_max_/*m* = ω, which is slightly larger than γ_max_/*m* = *ωk*_B_*T*/*E*_a_ but roughly in the same range.

For the brute-force TSH trajectory, we run 5 million time steps, and for the analysis, we define the lower state region *x* < −0.5 as the initial region *A* and the lower state region *x* > 0.5 as the final region *B*. A representative cutout of the TSH trajectory can be seen in Figure 4b and Figure S1a. It shows the typical behavior of a rare event, i.e., it oscillates for a long time in one of the two regions *A* or *B* before it undergoes a fast transition to the other region, where it oscillates again. As the diabatic coupling is very large, the crossing is strongly avoided and we expect small nonadiabatic effects. Indeed, the system spends only very short periods of time in the excited state, see orange circles in Figure 4b. The corresponding reaction rate constant for the transition from the initial region *A* to the final region *B* obtained with the brute-force TSH is (8.25 ± 0.28) × 10^–3^, see Table 1. This result nicely agrees with that obtained by the NAFFS simulation (8.72 ± 0.47) × 10^–3^, which aside marginal statistical deviations demonstrates the quantitative accuracy of our implementation. The computational details for the NAFFS simulation are given in Table S2.

**Table 1:** Rate Constants *k*_*AB*_, Number of Sampled Transition Paths, Mean Transition Times, Average Numbers of Hops in Adiabatic Representation, and Respective Standard Deviations (std) Obtained by Brute-Force TSH vs NAFFS Simulations on the Model Featuring an Avoided Crossing, with a Constant Coupling of *V*_c_ = 0.4.

	TSH	NAFFS
rate constant (10^–3^)	8.25 ± 0.28	8.72 ± 0.47
number of transition paths	1073	1805
mean transition time	2.2	2.1
std transition time	2.0	1.0
mean number of hops	0.0298	0.0321
std number of hops	0.2424	0.2515

Figure 4c and 4d shows the superimposed transition paths connecting the regions *A* and *B*, as obtained from TSH and NAFFS simulations. In contrast to the brute-force TSH simulation yielding a single long trajectory from which 1073 transition paths are cut out, the NAFFS simulation directly provides 1805 transition paths. The mean time that a transition path takes to go from *A* to *B* and the average number of hops occurring during the transition paths are also collected in Table 1, together with their corresponding standard deviations. The larger standard deviation for the transition time in TSH compared to NAFFS stems from the overall lower number of transition paths obtained with TSH and some outlying long transition paths among them. The good qualitative and quantitative agreement between NAFFS and TSH values confirms that the NAFFS simulation correctly samples transition paths.

**Figure 5:**
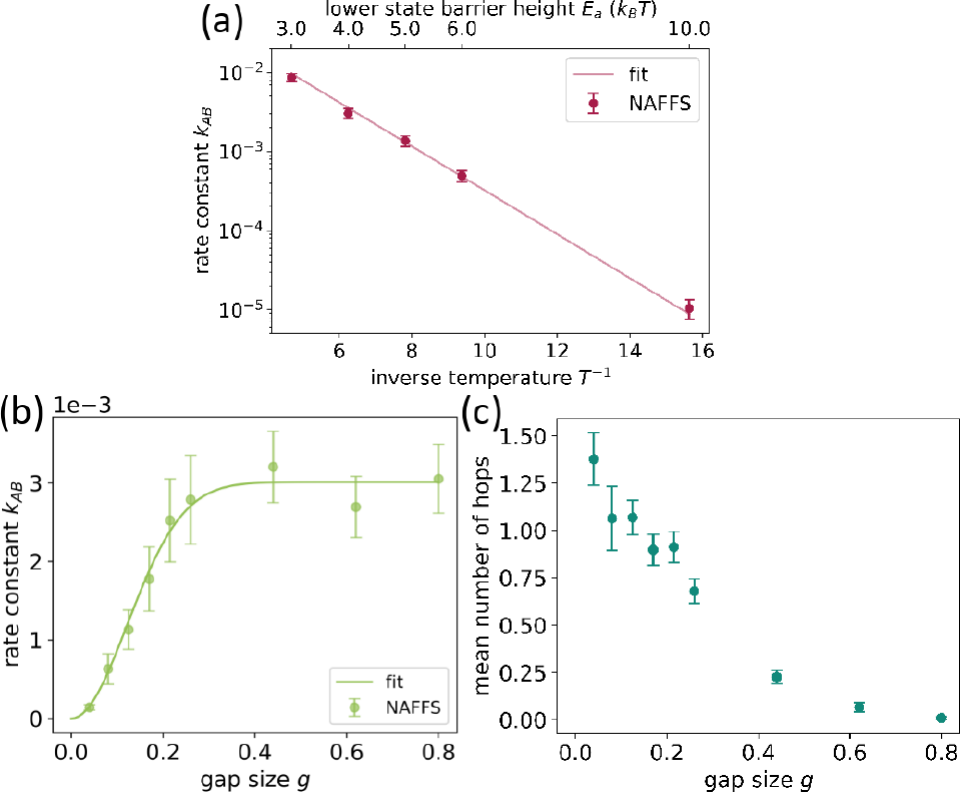
(a) Rate constant *k*_*AB*_ of the model system shown in Figure 4a with *g* = 0.8 as a function of the inverse temperature. Scale on the top shows the corresponding barrier height in units of *k*_B_*T*. Linear fit takes only the intercept as a fitting parameter, yielding ν = 0.19 (see eq 22). Error bars are plotted as 2σ confidence intervals. (b) Rate constant *k*_*AB*_ in the system shown in Figure 4a vs the adiabatic gap size *g* obtained from NAFFS calculations. Error bars are plotted as 2σ confidence intervals. Data are fitted according to eq 23, yielding *k*_*AB*_^0^ = 3.01 × 10^–3^ and *z* = 135.72. Analytical solution according to Landau–Zener theory is shown as gray dashed line. (c) Mean number of hops in the adiabatic representation vs adiabatic gap size as obtained from NAFFS transition paths. Obtained standard deviations of the mean are plotted as error bars.

In order to demonstrate the applicability of NAFFS in harsher conditions, we now decrease the temperature, which effectively increases the barrier and thus the rareness of the event. To avoid variations of the rate constant at different temperatures due to the definition of the boundaries of the stable regions *A* and *B*, we fix *A* as *x* < −1 and *B* as *x* > 1 on the lower state for the remaining simulations on this model. For the NAFFS simulations presented in what follows in section 4.1, we calculate the flux out of the initial region using the flux simulation method “flux with reset” mentioned in section 2.1. We expect the temperature dependence of calculated rate constants to follow Arrhenius’ law
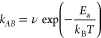
22with prefactor ν. The reaction rate constants obtained with NAFFS, shown in Figure 5a as a function of the inverse temperature, fit Arrhenius’ law remarkably well.

Table 2 illustrates the computational efficiency of NAFFS against TSH, showing that the speedup of NAFFS versus TSH increases with the rareness of the transition.

**Table 2:** Average Number of Time Steps Needed to Obtain One Transition Path in TSH and NAFFS (Flux Simulation Included) Calculations for Different Lower State Barrier Heights *E*_a_ (see Figure 5a)^a^

*E*_a_ (*k*_B_*T*)	TSH (time steps)	NAFFS (time steps)	speedup factor
3.0	2464	1083	2.3
4.0	7219	2459	2.9
5.0	16677	1750	9.5
6.0	38889	1120	34.7
10.0	226 465	1228	184.4

a The speedup factor in favor of NAFFS over TSH is also shown.

Note that the average number of time steps needed to obtain one NAFFS trajectory depends on the acceptance probabilities for the various interfaces (especially of the last shooting interface where the total number of final transition paths is determined) and, hence, on the choice of the interface placements. This explains that the average number of time steps required to obtain one reactive NAFFS path is always of the same order of magnitude, in stark contrast to the TSH simulations, which require an increasingly larger number of steps with decreasing temperature. Accordingly, for the lowest temperature (largest barrier height 10 *k*_B_*T*), NAFFS sampled 876 transition paths in about 1 million time steps (flux calculation included) whereas TSH sampled only 4 in the same amount of time steps. This implies a speed up of almost 200 in favor of NAFFS, i.e., a sampling acceleration of 2 orders of magnitude. We note that at this low temperature, an accurate rate constant calculation is no longer possible within reason with brute-force TSH but well feasible with NAFFS.

Finally, since the dynamics with *V*_c_ = 0.4 is predominantly adiabatic in the lower state potential, we investigate the effect of varying the diabatic coupling from 0.4 to 0.02, thus decreasing the diabatic gap from 0.8 to 0.04 and thus increasing the nonadiabaticity of the avoided crossing. The barrier height in terms of *k*_B_*T* is kept constant and equal to 4 *k*_B_*T* for all simulations. The rate constant is expected to decrease with smaller gaps, as the nonadiabatic effects increase, i.e., the number of hops in the adiabatic representation increases, and, thus, the probability of a transition for a trajectory that reaches the energy barrier is lower than that for systems with higher gap sizes. In other words, with smaller gaps, the nonadiabatic effects become more an obstacle that the system must overcome to complete a transition in addition to the potential energy barrier. In the limit of no diabatic coupling (*V*_c_ = 0 and, hence, *g* = 0), the system is diabatically trapped, i.e., the dynamics purely evolves on one diabatic state, and the transition rate constant is zero.

As expected, the calculated reaction rate constants are lower for smaller gap sizes, see Figure 5b. The probability that a trajectory coming from the initial region *A* hops to the excited state in the vicinity of the barrier, oscillates there for one period, and then falls back to the lower state in direction of *A* due to the inertia increases with decreasing gap size, i.e., the closer the adiabatic states come to each other. Accordingly, the mean number of surface hops in transition paths also increases with decreasing gap size, see Figure 5c.

We fitted the obtained rate constants (see Figure 5b) according to the Landau–Zener-type formula^105−107^
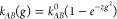
23where *k*_*AB*_^0^ is the lower state transition rate constant and *z* is a fitting parameter. This expression approximately describes the dependence of the reaction rate constant on the gap size.^99^ As shown in Figure 5b, the fit nicely reproduces our data. An analytical expression for the fitting parameter *z* can be obtained using Landau–Zener theory^106,107^ (see section S2.2.), which accurately matches our data (see Figure 5b, “Landau–Zener”). Some considerations regarding analytical expressions from Marcus theory^108^ in comparison to our data are given in section S2.2 and shown in Figure S1b in the SI. Even for small energy gap sizes and thus highly nonadiabatic situations, the NAFFS and TSH simulation rate constants agree (see Figure S1c), further validating our method.

### 4.2 Rare Event Dynamics through a Conical Intersection

**Figure 6:**
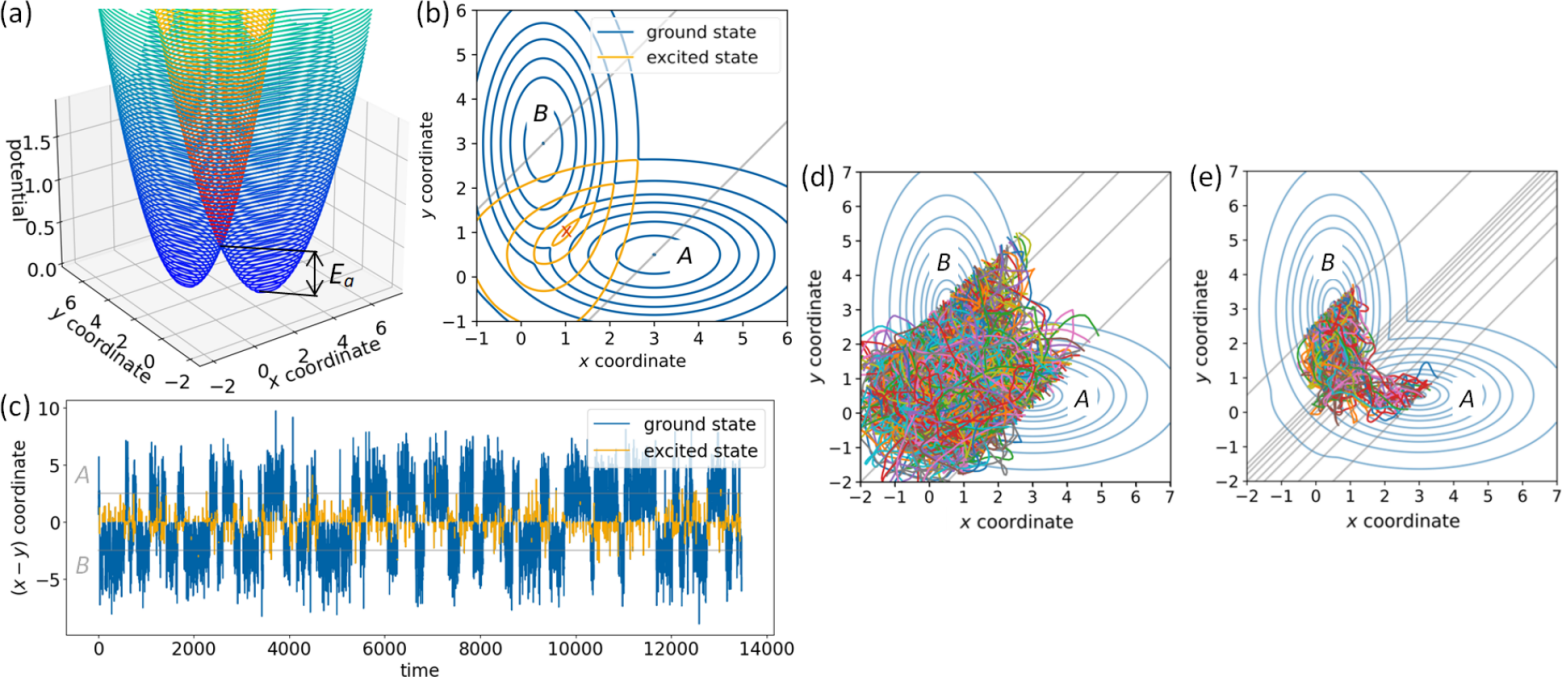
(a and b) Adiabatic representation of the potential energy landscape of eqs 24–26, shown as a function of *x* and *y* as a surface in three dimensions (a) and as a contour plot, where the boundaries of the initial and final regions *A* and *B* are shown in gray (b). Ground state is plotted in blue and excited state in orange. In b, the location of the conical intersection is marked in red. (c) Representative cutout of a TSH trajectory projected on (*x* – *y*) obtained for the model system as a function of time, where the first snapshot of the cutout is shifted to time zero for convenience. For better overview, stable state boundaries are shown in gray. Sojourns in the ground state are plotted in blue; stays in the excited adiabatic state are plotted in orange. (d and e) Transition paths obtained in the NAFFS simulation for temperatures chosen such that the ground-state barrier height equals 1 *k*_B_*T* (d) and 6 *k*_B_*T* (e). For the latter, the distribution is narrower than for the former, where higher energy regions of the PESs can be visited. Adiabatic ground-state contour lines are shown in blue and interfaces used in the NAFFS simulations in gray.

To examine the application of NAFFS to rare event dynamics in the vicinity of an explicit conical intersection, we consider a model with two coupled diabatic potential energy surfaces
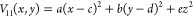
24and
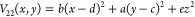
25with Cartesian coordinates *x*, *y*, and *z* and parameters *a* = 0.512, *b* = 0.128, *c* = 0.5, *d* = 3.0, and *e* = 12.8. The coupling between the two diabatic PESs is given by
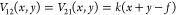
26with the prefactor *k* = 0.0128 and *f* = 2.3, resulting in the adiabatic PESs shown in Figure 6a and 6b. The narrow harmonic potential around the *z* coordinate allows us to consider the potential energy landscape as a function of two variables, *x* and *y*. As in section 4.1, we have weak normal mode coupling through velocity rescaling and decoherence. Again, all values are given in system-specific self-consistent units (see section S2.1 and Table S1), and the mass is *m* = 1.

We choose the default decoherence parameter^73^ (*C* = 0.1 in au, see section S2.3) and *s* = 25 substeps (see eq 11). As meaningful parameter values for the propagation using the Langevin thermostat, we choose a time step of 0.1348, a temperature *T* ≈ 0.6370, and a friction coefficient γ ≈ 0.7995. In contrast to the model discussed in section 4.1, here the rare event is not due to a high barrier, as this is only 1 *k*_B_*T*, but due to a small diabatic coupling that makes the system stay preferably on one *diabatic* surface (i.e., diabatic trapping). Accordingly, the dynamics show frequent hops between the ground and the excited adiabatic PESs in regions close to the conical intersection (see Figure 6c), i.e., in general twice during a typical oscillation period in one of the minima. Hence, in this case, the rare event is a rare passage from one *diabatic* state to the other, i.e., between the two diabatic PESs *V*_11_ and *V*_22_ (eqs 24 and 25). The initial region *A* and final region *B* are defined by (*x* – *y*) ≥ 2.5 and (*x* – *y*) ≤ – 2.5, respectively, plus the additional condition that the system needs to be located in the adiabatic ground state. For the NAFFS simulations presented in this, the stable region’s boundaries also correspond to the first and last interface, respectively, and we calculate the flux out of *A* using the “flux without reset” method discussed in section 2.1.

For this model, we performed a plain brute-force TSH simulation of 5 million time steps. The resulting rate constant, (5.58 ± 0.13) × 10^–3^, for the transition from *A* to *B* agrees very well with the rate constant obtained using a NAFFS calculation on the same model, (5.80 ± 0.30) × 10^–3^, performed with 1 million time steps in the flux simulation followed by 2000 shots per interface (see Table 3). Computational details for the NAFFS simulations are given in Table S3 of section S2.2. A second NAFFS simulation of one-half the size of the previous one (0.5 million time steps and 1000 shots per interface, see section S2.3) still yields the correct result, namely, *k*_*AB*_ = (5.56 ± 0.41) × 10^–3^, highlighting the efficiency of NAFFS.

**Table 3:** Rate Constant *k*_*AB*_, Number of Sampled Reactive Paths, Average Transition Times, Average Number of Hops in Adiabatic Representation, and Respective Standard Deviations (std) Obtained by Brute-Force TSH vs NAFFS Simulations for the Model with a Conical Intersection

	TSH	NAFFS
rate constant (10^–3^)	5.58 ± 0.13	5.80 ± 0.30
number of transition paths	1857	1025
mean transition time	87.67	87.98
std transition time	47.30	45.46
mean number of hops	2.89	2.73
std number of hops	2.10	2.13

**Figure 7:**
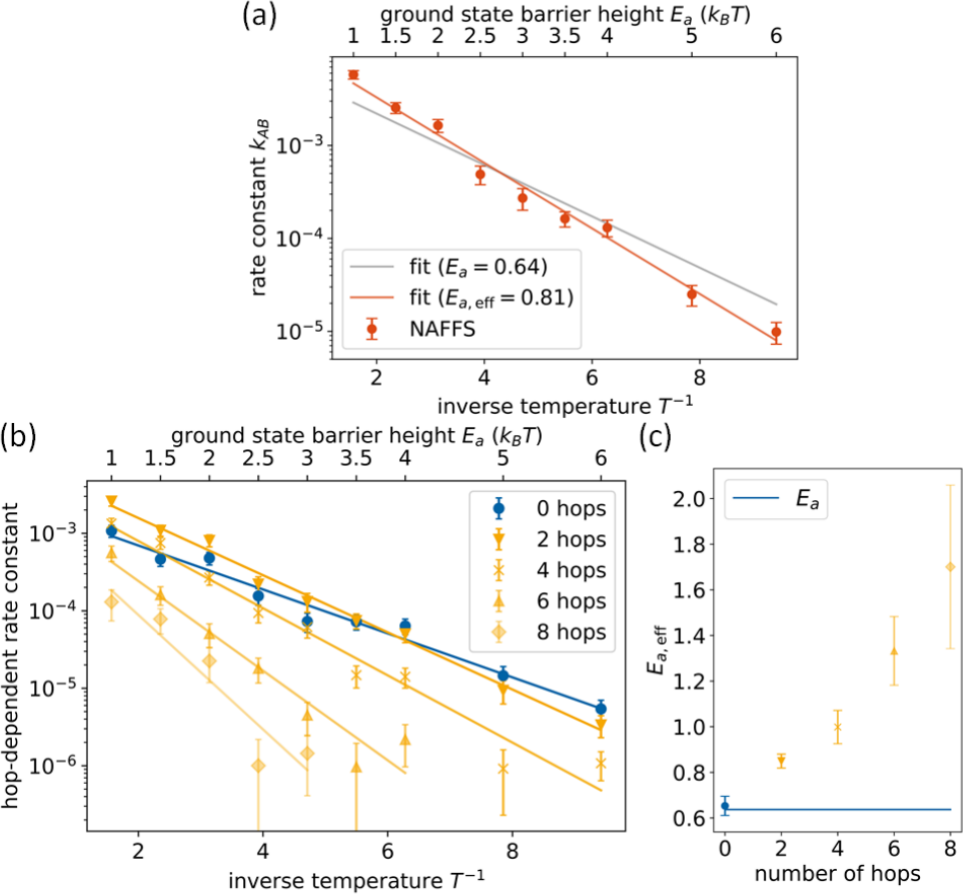
(a) Rate constant *k*_*AB*_ as a function of the inverse temperature (corresponding ground-state barrier in units of *k*_B_*T* is indicated on the upper axis). Error bars are plotted as 2σ confidence intervals. Data are fitted according to eq 27, yielding *E*_a,eff_ = 0.812 and ν = 0.017, and to eq 22, yielding ν = 0.0079. (b) Reaction rate constants due to transition paths from *A* to *B* that have a certain number of hops as a function of the temperature. Blue indicates a transition path purely in the adiabatic ground state (0 hops), and orange implies transition paths involving the excited state. Data are fitted according to eq 27. Data for more than 8 hops (see Figure S2c) are not statistically significant. (c) Effective activation energies as a function of the number of hops. Blue line indicates the ground-state energy barrier. Error bars are shown as 2σ confidence intervals.

The transition paths directly obtained by the NAFFS simulations (Figure 6d) are very similar to the ones cut out from the brute-force TSH trajectory (Figure S2a), demonstrating that NAFFS correctly samples transition paths in strong nonadiabatic regimes. Therefore, we next change the parameters of our model system to study it under different conditions. First, we investigate the dependence of the reaction rate constant on temperature. As can be seen in Figure 7a, due to the stronger nonadiabaticity of the system, the dependence is stronger than that in the case of the avoided crossing (recall Figure 5a), i.e., the slope in a log(*k*_*AB*_) vs *T*^–1^ plot is steeper than that given by Arrhenius’ law (eq 22) with an activation energy that equals the ground-state barrier height (see Figure 7a). Fitting the reaction rate constants with the expression
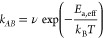
27with constant ν yields an effective activation energy of *E*_a,eff_ = 0.812 ± 0.036, which is significantly higher than the ground-state energy barrier of *E*_a_ = 0.64. This means that the nonadiabatic effects lead to an additional barrier that decreases the probability of the system to undergo a transition.

The obtained NAFFS transition paths show the expected qualitative behavior for different temperatures: their distribution is broader for higher temperatures as the system has more energy available and is narrower for low temperatures where the transition paths are located in the region around the conical intersection. In regions close to the conical intersection, the energy that the system needs to transition to the final region *B* is lower than that in regions far away from the conical intersection; see the narrower distribution of transition paths in Figure 6e along the *x* = *y* diagonal direction compared to Figure 6d. At low temperatures (see Figure 6e), the rare event is mainly determined by the high potential barrier, whereas for high temperatures (see Figure 6d), the rareness is predominantly caused by the nonadiabatic effects.

Figure 7b shows that the rate constants of reactive paths that exhibit a given number of hops between the ground and the excited adiabatic PESs differ from each other. In general, the fraction of transition paths that do not undergo hops increases with decreasing temperature, while the fraction of transition paths that feature hops decreases with decreasing temperature (see Figure S2b). This is because the system needs energy to undergo hops to the excited state but has less energy available the lower the temperature is (see Figure S2d). Note that due to using velocity rescaling (see eq 16), the number of hops to the excited state might be slightly overestimated because the system can use all of the kinetic energy along the velocity vector to perform a hop. Rescaling along the NAC direction,^46^ usually regarded as more accurate,^94^ would lead to fewer upward hops because less kinetic energy is usually available along the NAC direction. For simplicity, here we use velocity rescaling in all NAFFS and reference brute-force TSH simulations.

Since we defined the initial and final regions *A* and *B* in the adiabatic ground state, a transition path from *A* to *B* can only have an even number of hops in the adiabatic representation. We classify all obtained transition paths from Figure 7a according to the number of hops they undergo during their transition (“0 hops”, “2 hops”, “4 hops”, ...), and for each of these classes, we compute rate constants depending on the temperature (see Figure 7b). These “hop-dependent” rate constants (see Figure 7b) are fitted according to eq 27, yielding effective activation energies *E*_a,eff_ for transition paths with a certain number of hops between the ground and the excited adiabatic PESs. *E*_a,eff_ increases with increasing number of hops, which again agrees with the necessity to spend energy for hopping events (see Figure 7c). The effective activation energy for transition paths showing 0 hops, i.e., reaction paths evolving entirely in the ground state, is *E*_a,eff_ = 0.653 ± 0.043, which within the range of uncertainty aligns accurately with the ground-state energy barrier of *E*_a_ = 0.64. Hence, the fraction of transition paths evolving only in the ground state (“0 hops”) follows Arrhenius’ law even if the overall dynamics including all transition classes is highly nonadiabatic.

Furthermore, nonadiabatic transition paths also follow approximately Arrhenius’ law but with a higher effective activation energy. The latter can be understood when thinking about nonadiabatic effects as prolonging the transition path (e.g., because of turning around and having to come back again), which can be compensated by higher energies, and hence, the reaction barrier seems effectively higher.

An estimate of the computational savings obtained when using our NAFFS implementation is shown in Table 4. As in the case of the rare event dynamics through an avoided crossing (Table 2), here we also achieve a speedup factor of about 200 for the largest barrier height considered.

**Table 4:** Average Number of Time Steps Needed in an NAFFS Simulation to Obtain One Reactive Path (Flux Simulation Included) and Average Number of Time Steps Needed in a Brute-Force TSH Simulation to Obtain One Reactive Path, Shown for Different Ground-State Barrier Heights *E*_a_ (see Figure 7a)^a^

*E*_a_ (*k*_B_*T*)	TSH (time steps)	NAFFS (time steps)	speedup factor
1.0	2865	1165	2.5
1.5	5618	1040	5.4
2.0	12195	837	14.6
2.5	26316	850	31.0
3.0	66667	732	91.1
3.5	66667	1987	33.6
4.0	166 667	1495	111.5
5.0	1 000 000	8460	118.2
6.0	1 000 000	4968	201.3

a The speedup factor in favor of NAFFS over TSH is also shown.

## 5 Conclusions

In this work, we have introduced a nonadiabatic forward flux sampling (NAFFS) method that uses the trajectory surface hopping (TSH) algorithm for the underlying dynamics simulation. This method extends the previous fields of application of FFS to capture rare events in electronically excited systems, such as those initiated by the absorption of a photon. NAFFS is therefore suitable to deal with excited-state processes that occur on very long time scales, which cannot be otherwise accessed with plain brute-force TSH simulations. Using two models that exemplify different regimes of rareness and nonadiabaticity, we demonstrate that NAFFS produces quantitatively and qualitatively correct results at a computational cost that is 2 orders of magnitude lower than that of conventional TSH molecular dynamics simulations. Unlike previous efforts to develop nonadiabatic transition path sampling methods,^45^ our method does not need to propagate trajectories back in time and, hence, avoids serious problems in long simulations that include several hopping events.^44,47^

The presented approach is particularly promising to investigate photoinduced chemical reactions that are hindered by potential energy barriers or very small nonadiabatic couplings and thus take a long time to occur. Exciting examples include DNA damage and repair processes,^109,110^ enone [2 + 2] photocycloadditions,^111^ and many more.

## Supporting Information Available

Additional implementation details and code availability, reduced unit systems, NAFFS calculation details for avoided crossing and conical intersection systems, consideration of Landau–Zener and Marcus theory for the avoided crossing system, and analysis of transition paths for the conical intersection system (PDF)
