# Supplementary material for: Nonadiabatic Forward Flux Sampling for Excited-State Rare Events
Source: J Chem Theory Comput. 2023 Mar 1;19(6):1657–71. doi: 10.1021/acs.jctc.2c01088 (PMC10061683; doi:10.1021/acs.jctc.2c01088)
Supplement: Supplementary file 1 — ct2c01088_si_001.pdf [file ct2c01088_si_001.pdf]

Supporting Information: Nonadiabatic forward flux sampling for excited-state rare events

Madlen Maria Reiner,[†,‡] Brigitta Bachmair,[†,¶] Maximilian Xaver Tiefenbacher,[†,¶] Sebastian Mai,[§] Leticia González,[*,†,§] Philipp Marquetand,[*,†,§] and Christoph Dellago[*,†,∥]

†*Research Platform on Accelerating Photoreaction Discovery (ViRAPID), University of Vienna, Währinger Straße 17, 1090 Vienna, Austria*

‡*Vienna Doctoral School in Physics, University of Vienna, Boltzmanngasse 5, 1090 Vienna, Austria*

¶*Vienna Doctoral School in Chemistry, University of Vienna, Währinger Straße 42, 1090 Vienna, Austria*

§*Institute of Theoretical Chemistry, Faculty of Chemistry, University of Vienna, Währinger Straße 17, 1090 Vienna, Austria*

∥*Faculty of Physics, University of Vienna, Kolingasse 14-16, 1090 Vienna, Austria*

E-mail: leticia.gonzalez@univie.ac.at; philipp.marquetand@univie.ac.at; christoph.dellago@univie.ac.at

S1 Implementation details

S1.1 OPS (Open Path Sampling)

The modified OPS[1,2] version used in this work includes engines for SHARC[3] and SchNarc,[4] and an optimized engine for SchNarc called `SchNarcOpt`. Furthermore, we add a path

simulator called `forward_flux`, containing different versions of FFS (forward flux sampling)[5] algorithms, in particular a general one applicable to any engine, and optimized versions dedicated to the `SchNarcOpt` engine. The latter include a general one and one that can be restarted at each interface. The code is available at `https://github.com/MadlenReiner/openpathsampling/tree/virapid`.

S1.2 SHARC (Surface Hopping including ARbitrary Couplings)

We include a Langevin thermostat[6,7] in the SHARC program. The SHARC code including our modifications is available upon request and will be made publicly available in a forthcoming release.

S1.3 PySHARC (Python wrapper of SHARC)

PySHARC is an interface used to perform SHARC simulations in a Python program. We modified the function `run_sharc` within the class `SHARC_INTERFACE` so that it is possible to execute single time steps instead of a whole SHARC trajectory. The modified version of the code is available on request and will also be made publicly available in a forthcoming release of SHARC.

S1.4 SchNarc (Interface between SchNet and SHARC)

SchNarc[4] is originally designed as an interface between SHARC and an extension to excited-state properties of the neural network potential package SchNet.[8,9] The module `schnarc_md.py` is modified to incorporate the analytical models. Examples for the models discussed in the paper are provided at `https://github.com/MadlenReiner/SchNarc/tree/virapid/src/scripts`.

S2 Analytical model systems

S2.1 Reduced unit systems

Since our models are analytically constructed, using conventional units like femtoseconds for times or electron volts for energies is not the best solution, because it may suggest connections to real chemical systems that do not exist. Therefore, we decided to convert our results to system-specific units that are meaningful for the respective analytical models and that are internally consistent, *i.e.*, comparable relative to each other within each system of units.

For the model featuring an avoided crossing (see Sec. 4.1), we choose our energy unit $\widehat{=}$ 1 ϵ and length unit x_0 such that the diabatic potential energy surfaces in x are defined as

$$V_{\pm} = \frac{\epsilon}{x_0^2}(x - x_0)^2 \tag{1}$$

i.e., the two diabatic PESs have minima at $x = \pm 1\ x_0$ and intersect the y axis at 1 ϵ. Masses are measured in units of the mass m of the system. Times are measured in units of $\sqrt{\frac{mx_0^2}{\epsilon}}$. The unit of action in our unit system is $\sqrt{\epsilon m x_0^2}$. The temperature in a conventional unit system is accessed via the relation $\frac{k_B T}{\epsilon} = 1$.

For the model featuring a conical intersection (see Sec. 4.2), we choose energy, length, time, and mass units of our unit system in the same manner, by placing our diabatic PESs minima at coordinates $(x, y) = (0.5\ x_0, 3.0\ x_0)$ and $(x, y) = (3.0\ x_0, 0.5\ x_0)$, and fixing one energy unit ϵ as intersect of the diabatic potentials and the z axis.

The final conversion table between the unit system used by SHARC input files (*i.e.*, atomic units for everything except for time that is measured in femtoseconds) and the unit systems used for the analytical model systems are shown in Table S1.

Table S1: Unit conversion table for the analytical model systems between conventional units and system-specific self-consistent units used in this work

	Model system "avoided crossing"	Model system "conical intersection"
energy E	$[E] = 1\ \epsilon \mathrel{\widehat{=}} 0.05$ Ha	$[E] = 1\ \epsilon \mathrel{\widehat{=}} 0.078125$ Ha
length L	$[L] = 1\ x_0 \mathrel{\widehat{=}} 1$ Bohr	$[L] = 1\ x_0 \mathrel{\widehat{=}} 1$ Bohr
mass M	$[M] = 1\ m \mathrel{\widehat{\approx}} 1837.15$ m$_e$	$[m] = 1\ m \mathrel{\widehat{\approx}} 1837.15$ m$_e$
time t	$[t] = 1\ \sqrt{\frac{mx_0^2}{\epsilon}} \mathrel{\widehat{\approx}} 4.6366$ fs	$[t] = 1\ \sqrt{\frac{mx_0^2}{\epsilon}} \mathrel{\widehat{=}} 3.7093$ fs
temperature T	$[T] = 1\ \frac{\epsilon}{k_B} \mathrel{\widehat{\approx}} 15788.76$ K	$[T] = 1\ \frac{\epsilon}{k_B} \mathrel{\widehat{\approx}} 24669.93$ K
Planck's constant $\hbar$	$\hbar \approx 0.1043379668\ \sqrt{\epsilon m x_0^2}$	$\hbar \approx 0.0834703735\ \sqrt{\epsilon m x_0^2}$

S2.2 Rare event dynamics through an avoided crossing

The time step used in this model system is 0.25 fs in conventional units. We use a TSH decoherence parameter $C = 2$, which in atomic units corresponds to 0.1 Ha.[10] Computational details for the NAFFS simulation given in Table 1 in our paper are shown in Table S2. The flux is calculated using the flux calculation method "flux without reset" mentioned in Sec. 2.1 in our paper.

Table S2: Computational details for the NAFFS simulation presented in Table 1 in our paper.

flux sim.	time steps	crossing events	flux (10^{-3})
	1 million	2137	(77.1 ± 1.7)
NAFFS sim.	interfaces in x	shots per interface	crossing prob.
	$-0.5 \to -0.2$	2000	$(24.6 \pm 1.0)\%$
	$-0.2 \to 0.0$	2000	$(50.9 \pm 1.2)\%$
	$0.0 \to 0.5$	2000	$(90.25 \pm 0.67)\%$

For readers interested in energy difference *vs.* time plots instead of coordinate *vs.* time plots (Fig. 4b of main text), we show this figure as energy difference *vs.* time version in Fig. S1(a). However, since the PESs are symmetric around $x = 0$, one cannot distinguish initial region A and final region B in such a plot –an aspect highly relevant for our focus on sampling transition paths.

In Landau-Zener theory, the expression for the parameter z (see Eq. (22) in our paper)

is given by[11,12]

$$z = \frac{2\pi}{\hbar v |F_2 - F_1|} \tag{2}$$

with velocity v, and forces F_1 and F_2 acting upon the two states. Analytically, we diagonalize the diabatic Hamiltonian to get analytic expressions for the adiabatic potentials. Using their derivatives, we obtain for our models $|F_2 - F_1| = 4$. Further, we obtain

$$v = \sqrt{\frac{k_B T}{m}} = \sqrt{\frac{1}{4} \cdot \left(1 + \frac{1}{4} \cdot V_c^2 - V_c\right)} \tag{3}$$

with parameters as explained in the manuscript. The gap size is $g = 2V_c$, and we are using $T = \frac{E_a(g)}{4k_B}$. The respective plot is shown in Fig. 5b, "Landau-Zener"). It perfectly matches our data.

In the following, we give some considerations regarding analytical results for Fig. 5b from Marcus theory. The full rate equation according to Marcus theory[13] is

$$k_{AB} = \frac{2\pi}{\hbar} \frac{|V_c|^2}{\sqrt{4\pi\lambda k_B T}} \exp\left(-\frac{(\lambda + \Delta G)^2}{4\lambda k_B T}\right). \tag{4}$$

In our case, the "reorganization energy" is $\lambda = 4\epsilon$ and the free energy difference is $\Delta G = 0$. Parameters are as explained in our manuscript. Thus,

$$k_{AB} = \frac{2\pi}{\hbar} \frac{|V_c|^2}{\sqrt{16\pi\epsilon k_B T}} \exp\left(-\frac{\epsilon}{k_B T}\right). \tag{5}$$

This is an approximation for small gap sizes (nonadiabatic limit) and in this region, matches our data within the statistical deviations (see Fig. S1b, blue dashed line).

Brute-force TSH results accompanying Fig. 5b in our paper are shown in green in Fig. S1c. They agree very well with our NAFFS results.

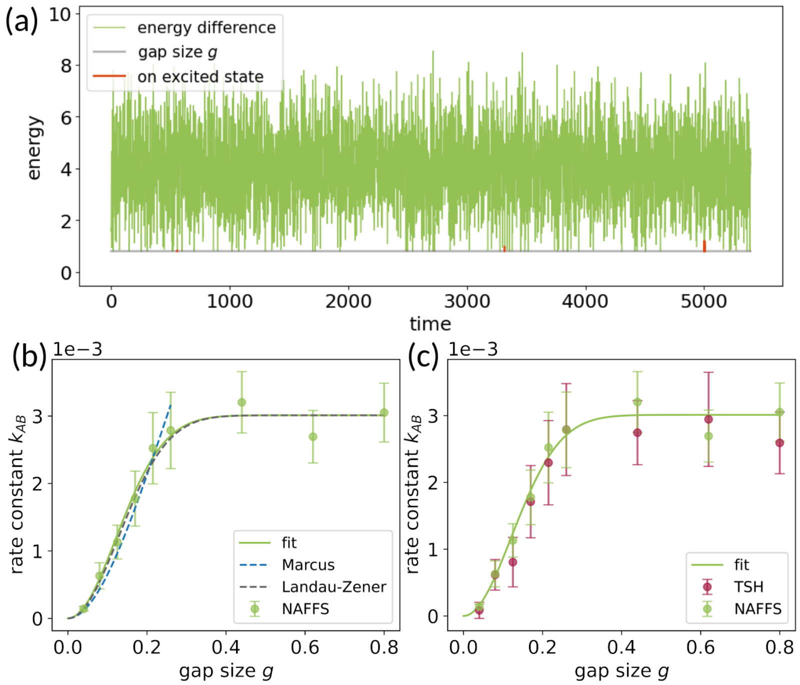

Figure S1: (a) Representative cutout of a TSH trajectory (see Fig. 4b in the paper) as an energy difference *vs.* time plot, where the first snapshot of the cutout is shifted to time zero for convenience. Trajectory parts that are spent in the excited state are shown in red.(b) Fig. 5b with additional Marcus results plotted as blue dashed line. (c) Brute-force TSH rates as a function of the gap size g compared to Fig. 5b in our paper.

S2.3 Rare event dynamics through a conical intersection

The time step used in this model system is 0.5 fs in conventional units. We use a decoherence parameter $C = 1.28$, which in atomic units corresponds to 0.1 Ha.[10]

Computational details for the NAFFS simulation given in Table 3 in our paper are shown in Table S3. For the NAFFS simulation of half the size that is mentioned in the paper, we use 0.5 million time steps in the flux calculation and 1000 shots per interface, and the definition of the interfaces is not changed compared to Table S3.

Table S3: Computational details for the NAFFS simulation presented in Table 3 in the main paper.

flux sim.	time steps	crossing events	flux (10^{-3})
	1 million	6527	(100.1 ± 2.8)
NAFFS sim.	interfaces in $(x - y)$	shots per interface	crossing prob.
	$2.5 \to 0.0$	2000	$(29.9 \pm 1.1)\%$
	$0.0 \to -1.5$	2000	$(37.8 \pm 1.1)\%$
	$-1.5 \to -2.5$	2000	$(51.3 \pm 1.2)\%$

For qualitative comparison of the reactive paths of the TSH and NAFFS comparisons presented in our work, we show here (see Fig. S2a) the transition paths obtained in the TSH simulation consisting of 5 million time steps that is described in the paper (see Sec. 4.2). They qualitatively agree with the ones sampled in the NAFFS simulation, which are shown in Fig. 6d in our paper. Note that almost twice as many reactive paths are plotted in case of the TSH simulation (1857, see Fig. S2a) than by the NAFFS simulation (1025, see Fig. 6d in the paper), resulting in the TSH paths being denser. Note that the initial and final regions A and B are defined not only geometrically as plotted in Fig. S2a, but also in terms of the PES, namely on the ground state. Accordingly, paths that seem to sojourn in A or B in the plot are located on the excited state – and, hence, neither in A nor in B.

An evaluation of the average number of hops in transition paths as a function of the inverse temperature shows a decreasing behavior (see Fig. S2b). In accordance to that we find that the fraction of paths which undergo the transition purely on the ground state with respect to the total number of reactive paths increases with decreasing temperature, whereas

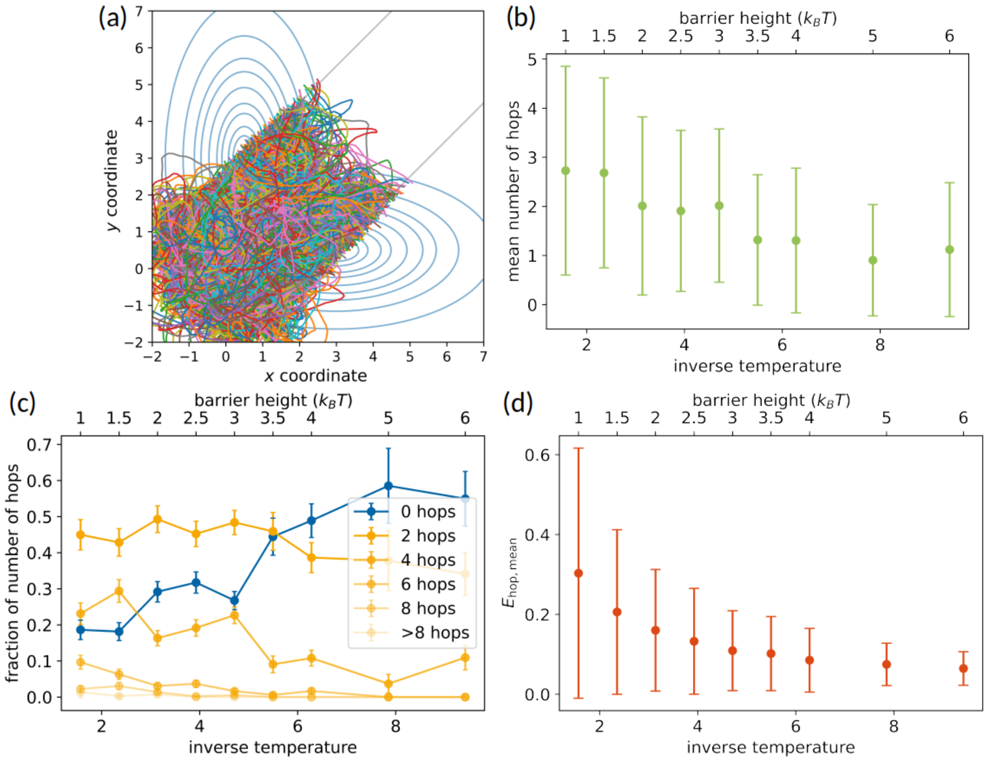

Figure S2: (a) 1857 transition paths obtained in the TSH simulation of the two-dimensional analytical model. Adiabatic ground state contour lines are shown in blue, and the boundaries of the initial and final regions are shown in gray. (b) The mean number of hops per NAFFS transition path as a function of the inverse temperature, and, alternatively, as a function of the barrier height. The error bars show the obtained standard deviation and do not indicate the accuracy of the obtained results. (c) The ratio of NAFFS transition paths showing a certain number of hops, where the x axes are the same as in (b). The values of the error bars Δf are calculated with respect to the number of paths N showing the specific number of hops and their ratio f with respect to the total number of transition paths, *i.e.*, $\Delta f = f \cdot \frac{\sqrt{N}}{N}$. (d) The average potential energy of a hop from the ground state to the excited state in a TSH simulation, where the x axes equal the ones in (b). The error bars indicate the obtained standard deviation.

the ratio of transition paths that show hops to the excited state decreases with decreasing temperature (see Fig. S2c). Fig. S2 also shows the ratio of reactive paths that have more than eight hops in a transition. Only in the three calculations with the highest temperatures this fraction is greater than zero. Where such hops occur, their number is very small (only 29 hops in all calculations combined), both of which militate against examining these numbers in more detail statistically as they are not significant, so we limit ourselves to the paths that show eight hops or fewer.

Fig. S2d shows the mean energy of a hop from the ground state to the excited state as a function of the inverse temperature. It is obtained as the potential energy difference at the geometries where the hops occur. As expected, it decreases with decreasing temperature, since energy must be spent for hops, and the system has less energy available at a lower temperature. At higher temperature, both hops that require a lot of energy and those that cost little energy can occur, while at low temperature, only those hops that require little energy are possible. Accordingly, the standard deviation of the mean energy decreases with decreasing temperature.

References

(1) Swenson, D. W.; Prinz, J. H.; Noe, F.; Chodera, J. D.; Bolhuis, P. G. OpenPathSampling: A Python Framework for Path Sampling Simulations. 1. Basics. *J. Chem. Theory Comput.* **2019**, *15*, 813–836.

(2) Swenson, D. W.; Prinz, J. H.; Noe, F.; Chodera, J. D.; Bolhuis, P. G. OpenPathSampling: A Python Framework for Path Sampling Simulations. 2.Building and Customizing Path Ensembles and Sample Schemes. *J. Chem. Theory Comput.* **2019**, *15*, 837–856.

(3) Mai, S.; Richter, M.; Heindl, M.; Menger, M. F.; Atkins, A.; Ruckenbauer, M.; Plasser, F.; Ibele, L. M.; Kropf, S.; Oppel, M.; Marquetand, P.; González, L. SHARC

2.1: Surface Hopping Including Arbitrary Couplings – Program Package for Non-Adiabatic Dynamics. 2019; `sharc-md.org`.

(4) Westermayr, J.; Gastegger, M.; Marquetand, P. Combining SchNet and SHARC: The SchNarc Machine Learning Approach for Excited-State Dynamics. *J. Phys. Chem. Lett.* **2020**, *11*, 3828–3834.

(5) Allen, R. J.; Warren, P. B.; Ten Wolde, P. R. Sampling rare switching events in biochemical networks. *Phys. Rev. Lett.* **2005**, *94*, 018104.

(6) Grønbech-Jensen, N.; Farago, O. Research article : A simple and effective verlet-type algorithm for simulating langevin dynamics. *Mol. Phys.* **2013**, *111*, 983–991.

(7) Grønbech-Jensen, N.; Hayre, N. R.; Farago, O. Application of the G-JF discrete-time thermostat for fast and accurate molecular simulations. *Comput. Phys. Commun.* **2014**, *185*, 524–527.

(8) Schütt, K. T.; Kindermans, P. J.; Sauceda, H. E.; Chmiela, S.; Tkatchenko, A.; Müller, K. R. SchNet: A continuous-filter convolutional neural network for modeling quantum interactions. *Adv. Neural Inf. Process. Syst.* **2017**, *30*, 992–1002.

(9) Schütt, K. T.; Sauceda, H. E.; Kindermans, P. J.; Tkatchenko, A.; Müller, K. R. SchNet - A deep learning architecture for molecules and materials. *J. Chem. Phys.* **2018**, *148*, 241722.

(10) Granucci, G.; Persico, M.; Zoccante, A. Including quantum decoherence in surface hopping. *J. Chem. Phys.* **2010**, *133*, 134111.

(11) Landau, L. D. A theory of energy transfer II. *Phys. Z. Sowjetunion* **1932**, *2*, 118.

(12) Zener, C. Non-adiabatic crossing of energy levels. *Proc. R. Soc. London, Ser. A* **1932**, *137*, 696–702.

(13) Taylor, N. B.; Kassal, I. Generalised Marcus theory for multi-molecular delocalised charge transfer. *Chem. Sci.* **2018**, *9*, 2942–2951.
